# Immunoglobulin E-Dependent Activation of Immune Cells in Rhinovirus-Induced Asthma Exacerbation

**DOI:** 10.3389/falgy.2022.835748

**Published:** 2022-02-22

**Authors:** Toshiaki Kawakami, Kazumi Kasakura, Yu Kawakami, Tomoaki Ando

**Affiliations:** ^1^Laboratory of Allergic Diseases, Center for Autoimmunity and Inflammation, La Jolla Institute for Immunology, La Jolla, CA, United States; ^2^Department of Dermatology, School of Medicine, University of California, San Diego, La Jolla, CA, United States; ^3^Atopy (Allergy) Research Center, Juntendo University Graduate School of Medicine, Tokyo, Japan

**Keywords:** asthma, rhinovirus, mast cells, dendritic cells, IgE, omalizumab

## Abstract

Acute exacerbation is the major cause of asthma morbidity, mortality, and health-care costs. Respiratory viral infections, particularly rhinovirus (RV) infections, are associated with the majority of asthma exacerbations. The risk for bronchoconstriction with RV is associated with allergic sensitization and type 2 airway inflammation. The efficacy of the humanized anti-IgE monoclonal antibody omalizumab in treating asthma and reducing the frequency and severity of RV-induced asthma exacerbation is well-known. Despite these clinical data, mechanistic details of omalizumab's effects on RV-induced asthma exacerbation have not been well-defined for years due to the lack of appropriate animal models. In this Perspective, we discuss potential IgE-dependent roles of mast cells and dendritic cells in asthma exacerbations.

## Introduction

Asthma is a T helper cell 2 (Th2) cell-driven chronic inflammatory lung disease, characterized by airway inflammation, airway hyperresponsiveness (AHR), airway remodeling, and reversible airway obstruction (1, 2). The prevalence of asthma has been increasing for the last several decades (3). Five-10% of the patients have severe asthma, which is often difficult to treat (4). No treatment is curative, and existing drugs are often ineffective in controlling symptoms (5, 6). RV infection is associated with the majority of asthma exacerbations (7–12), whereas respiratory syncytial virus (RSV) is more associated with non-allergic asthma (11, 13). The risk for wheezing with RV is associated with allergic sensitization (e.g., HDM-specific IgE) and type 2 airway inflammation (11–17). Some, but not all, studies (18–21) suggest that impaired type I and III interferon (IFN) responses to RV infection may contribute to asthma exacerbation. RV infection also induces production of IL-25 (22), IL-33 (23), thymic stromal lymphopoietin (TSLP) (24), and granulocyte macrophage colony-stimulating factor (GM-CSF) (25) in lung epithelial cells, promoting type 2 airway inflammation (26–29). Clinical evidence supports the pathogenic role for IgE and mast cells in asthma and RV-induced asthma exacerbation: mast cells are increased in the airway epithelium (30) and within the smooth muscle layer (31–33) in allergic asthma, and in the alveolar parenchyma of uncontrolled allergic asthma (34). An increased percentage of degranulated mast cells are found in the mucous glands from fatal asthma (35). Unlike the Th2-low group, the Th2-high group of asthmatics highly express Th2 (*IL4, IL5, IL13*; IL-13-regulated genes, *POSTN, CLCA1, SERPINB2*) and mast cell (*TPSB2, TPSAB1*, and *CPA3*) genes in the airway epithelium (36–38), consistent with the recruitment of mast cells to the airway epithelium from the submucosa (39, 40). Mast cells are also recruited to the bronchial epithelium following RV infection (41). RV can replicate in mast cells and induce their activation (42–44). Importantly, omalizumab is indicated for moderate-to-severe asthma (45–48) and reduces RV-induced asthma exacerbation (47, 49, 50).

Despite these data supporting the pathogenic role for IgE and mast cells in asthma, the study of this topic has a twisted history. Studies in 1990s raised mixed results, with some supporting their role (51, 52), but others revealing the lack of their role in *in vivo* asthma models (51, 53–61). This confusion was resolved by two studies published in year 2000 showing that the contribution of mast cells to airway inflammation can be seen in mice sensitized with small amounts of a conveniently available antigen [i.e., ovalbumin (OVA)] without the strong Th2-skewing adjuvant alum (62, 63). However, the situation became murky again when clinically relevant allergens such as house dust mite (HDM) were increasingly used. Numerous studies have been conducted on HDM-induced airway inflammation in murine models. While most studies failed to mention on the role of IgE or mast cells (64–79), a recent study showed that HDM-induced airway inflammation is not dependent on IgE or FcεRI (80). In this Perspective, we will overview fundamental aspects of IgE, IgE receptors, and mechanisms of anti-IgE mAb's function. Then, we will describe cell types that are targeted by anti-IgE mAb-mediated protection against asthma exacerbations. Finally, we will discuss animal models of asthma and RV-induced asthma exacerbation, which will potentially solve the current enigma of how RV induces asthma exacerbations. Our discussion of IgE-related studies will be limited to those required for understanding the topic. Those who want to have an updated deeper understanding are referred to recent excellent reviews (81–87).

### IgE, IgE Receptors, and Omalizumab

IgE is the least abundant immunoglobulin in serum. Multiple mechanisms control IgE levels from its synthesis to degradation: low efficiency of class-switch recombination to IgE, lower surface expression of membrane IgE (mIgE) than of mIgG1 on germinal center (GC) B cells, and increased apoptosis of IgE^+^ GC B cells. Recent studies on several reporter mice and GC reaction-mimicking B cell cultures (iGB cells) on 40LB feeder cells showed that IgE-producing B cells swiftly exit GCs and differentiate into plasma cells (PCs) and that IgE-producing GC cells die by apoptosis (88–91). A recent study found that IgE-BCR without antigen stimulation induces PI3K-mediated mTOR activation that increases IRF4 protein (not transcription), leading to IgE^+^ PC differentiation, and that chronic calcium signaling in IgE^+^ B cells and PCs culminates in apoptosis (92). Therefore, IgE^+^ memory B cells and IgE^+^ PCs are scarce. High-affinity IgE is produced by consecutive class-switch recombinations from IgM to IgG to IgE, whereas low-affinity IgE is produced by a direct switch from IgM to IgE (93). IgE production is induced by IL-4 and IL-13 (94, 95). T follicular helper (Tfh) cell-derived IL-4 is necessary for IgE production (96). Recently, a rare population of IL-13-producing Tfh cells (Tfh13) was shown to be required for production of high-affinity IgE (97). The half-life of infused IgE in the serum is very short (2 days) compared to IgG (18–23 days) (98). Several mechanisms likely contribute to the short half-life of IgE: rapid removal of free IgE from the circulation by binding to mast cells and basophils; degradation of IgE by extravascular and membrane-bound proteases; binding of IgE glycans to lectins leading to delivery for degradation; receptor-mediated endocytosis; and digestion of IgE in endolysosomes caused by lack of protection by FcRn.

There are two IgE receptors, the high-affinity receptor FcεRI and the low-affinity receptor CD23 (FcεRII). FcεRI expressed on mast cells and basophils is a heterotetramer of three subunits, an IgE-binding α subunit, a receptor-stabilizing and signal-amplifying β subunit, and signal-initiating disulfide-linked two γ subunits (99). IgE binds FcεRI at 1:1. While FcεRI expression is limited to mast cells and basophils in mice under homeostatic conditions, FcεRI in humans is additionally expressed by eosinophils, DCs and Langerhans cells. FcεRI is also expressed by bronchial epithelial cells in some asthmatics (100). The non-mast cell/basophil FcεRI consists of αγ_2_ heterotrimers. It has been known for long that levels of FcεRI expression on the cell surface correlates well-with serum levels of IgE (101, 102). This seems to be due to stabilization of FcεRI by bound IgE (103, 104). Thus, reduction in IgE levels (e.g., by omalizumab) will reduce the cell surface expression of FcεRI (105). Binding of a multivalent antigen to the mast cell and basophil surface IgE induces aggregation or crosslinking of FcεRI that triggers the activation of complex signaling events, eventually resulting in degranulation, eicosanoid synthesis and release, and cytokine production and secretion. These events cause allergic reactions ranging from local redness and itch to lethal systemic anaphylaxis.

CD23 is a type 2 transmembrane protein that forms a homotrimer composed of an IgE-binding C-type lectin head, a long α-helical coiled-coil stalk, a transmembrane domain and an N-terminal short cytoplasmic portion. CD23 is expressed on B cells, monocytes, DCs, Langerhans cells, eosinophils, and respiratory and gastrointestinal epithelial cells (106). Antigen presentation by CD23-bound IgE is known as IgE-mediated facilitated antigen presentation that amplifies Th2 responses upon re-exposure to the same antigen. In this phenomenon, IgE-antigen complexes are transported to the spleen by recirculating CD23^+^ B cells where they are delivered to CD8α^−^ conventional DCs (cDCs) which induce proliferation of CD4^+^ T cells (107). CD23 expression by B cells is involved in regulation of IgE synthesis: engagement of CD23 with IgE suppresses IgE production and CD23-deficient mice exhibit stronger and long-lasting IgE response upon immunization (108). Conversely, CD23 transgenic mice exhibit decreased IgE production (109, 110). CD23-blocking mAb lumiliximab lowers IgE levels in humans (111). In contrast, soluble CD23 fragments promote IgE synthesis. Although CD23 plays a role in OVA-induced airway inflammation (112) by transcytosis of IgE immune complexes by lung epithelial cell CD23 (113), little is known about the role of CD23 in asthma exacerbations.

Omalizumab is a humanized IgG1κ monoclonal antibody (mAb) that binds to free human IgE and to membrane-bound form of IgE (mIgE) on the surface of B cells (114). Omalizumab inhibits IgE interactions with FcεRI and CD23 (115). As it binds to free IgE, omalizumab lowers free IgE levels and thus downregulates FcεRI levels on basophils and mast cells, limiting the degree of release of allergic mediators. Owing to these properties, omalizumab is indicated for the treatment of moderate-to-severe asthma (116), allergic rhinitis (117), and chronic idiopathic/spontaneous urticaria (CIU/CSU) (118–120). However, a newer anti-IgE mAb ligelizumab (121) failed to show its efficacy in the treatment of severe asthma (122), while it was more effective than omalizumab in the treatment of CIU/CSU (123). It was speculated that the differential efficiencies of omalizumab and ligelizumab in inhibiting FcεRI and CD23 bindings may contribute to the difference in the efficacies on severe asthma, although it requires further investigation.

### Dendritic Cells

Children with severe asthma are susceptible to respiratory virus-induced asthma exacerbations, particularly those with high serum IgE levels (16, 124). IFNs secreted by plasmacytoid DCs (pDCs) are essential for the host defense to viral infections. pDCs express FcεRI and its expression is controlled by IgE levels. Enhanced FcεRI expression in asthma inhibits virus-induced IFN-α and IFN-λ1 responses of human pDCs (19, 125, 126). Omalizumab treatment of asthmatics reduces FcεRI expression on the pDC surface and increases RV-induced pDC IFN-α responses (19, 125–127). Studies demonstrated a counterregulatory mechanism between FcεRIα and TLR7, by which expression of these proteins is inversely proportional (19, 125). These observations can potentially explain the PROSE data that preventive administration of omalizumab could dampen the seasonal increase in asthma exacerbations among school children (50). Another DC subset might be involved in respiratory virus-induced asthma exacerbations. Sendai virus (SeV) infection in mice results in cysteinyl leukotrienes-induced recruitment and survival of CD49d^+^ neutrophils and subsequent enhancement of FcεRI expression on lung cDCs. Meanwhile anti-SeV IgE antibodies are produced, and these IgE antibodies stimulate the lung cDCs to produce CCL28 via FcεRI. CCL28 recruits IL-13-producing Th2 and type 2 innate lymphoid cells (ILC2) (11, 128). Thus, cDCs might also be targeted by omalizumab.

**Figure 1:**
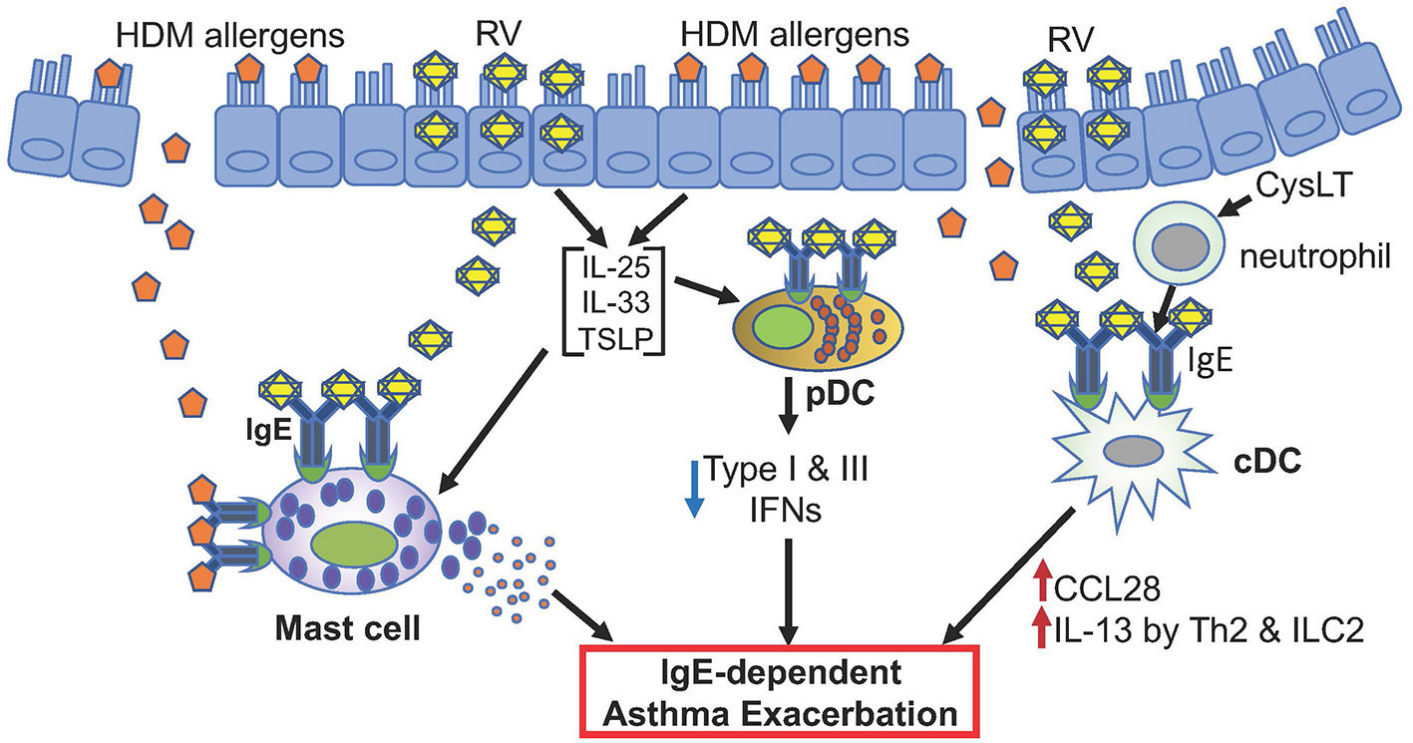
Hypothetical model of RV-induced asthma exacerbation. Only potential events involving IgE and FcεRI are depicted. Allergens such as HDM and rhinovirus antigens likely activate mast cells. pDCs highly expressing FcεRI in asthmatics may produce reduced levels of type I and type III IFNs when infected with rhinovirus. cDCs with high FcεRI expression may produce CCL28 that recruits IL-13-producing Th2 and ILC2 cells. These different cells likely contribute to RV-induced asthma exacerbation.

The clinical observations described above, particularly efficacy of omalizumab in reducing asthma exacerbations (49, 50, 116, 127, 129), strongly support the pathogenic role of IgE-bound mast cells, pDCs, and cDCs to RV-induced asthma exacerbation (Figure 1).

### Mast Cells and IgE in HDM-Induced Models of Asthma

Early studies of animal models of asthma mostly used chicken OVA as a model allergen. More recently, numerous studies used HDM for sensitization and challenge purposes, as up to 90% of asthmatics are sensitized with HDM. When mice were repeatedly exposed to intranasal injections of a single allergen such as HDM, ragweed and Aspergillus, tolerance was induced (66). In contrast, sensitization to double and triple allergens broke through tolerance and caused AHR, eosinophilic inflammation, mast cell and smooth muscle hyperplasia, mucus production, and airway remodeling. Mucosal exposure to triple allergens in the absence of an adjuvant induced chronic airway inflammation. Anti-IL-5 and anti-IL-13 antibodies inhibited inflammation and AHR in the acute asthma model but not in the chronic triple-allergen model (66). A similar procedure of intranasal sensitization/challenge with the above triple allergens showed that airway epithelial cell-derived colony stimulating factor (CSF1/M-CSF) had a critical role in the production of allergen specific-IgE and regulated the recruitment of alveolar DCs and enhanced the migration of cDC2s to the draining lymph node, leading to Th2 cell-mediated allergic lung inflammation (69).

In typical airway inflammation experiments using HDM, mice are sensitized with a commercial HDM via the intranasal or intratracheal route and then challenged with the same allergen via the same route. One day or a few days after the last allergen challenge, bronchoalveolar lavage fluids (BALF) are collected. Leukocytes (e.g., increased eosinophils, neutrophils, macrophages, lymphocytes) and cytokines (e.g., increased mRNAs for and/or proteins of IL-4, IL-5, IL-13) in BALF and lungs are reported together with total or allergen-specific IgE and IgG and lung functions (usually showing methacholine-induced increase in lung resistance). Remodeling such as goblet cell metaplasia is also reported. Acute and chronic disease-mimicking procedures were developed. Involvement of specific molecules or cells in these airway inflammation models has been defined using pharmacological and immunological reagents as well as genetically engineered mice lacking these molecules or cell types. For example, antibodies against GM-CSF (64) or chemical antagonists against TLR4 (67) reduced Th2 phenotype and AHR. Using bone marrow chimeras, Hammad et al. showed that TLR4 expression on radioresistant lung structural cells, but not on DCs, is necessary and sufficient for DC activation in the lung and for priming of effector T helper responses to HDM (67). TLR4 triggering on structural cells caused production of the innate proallergic cytokines TSLP, GM-CSF, IL-25 and IL-33.

Intranasal administration of a high dose of HDM for 5 days/week over 3 weeks induced allergic airway inflammation with mast cell expansion and HDM-induced bronchoconstriction, which was abrogated in mast cell-deficient Kit^W−sh/W−sh^ mice (130). In another HDM model, WT mice showed allergic airway inflammation with increased tryptase in BALF, but Kit^W−sh/W−sh^ mice showed a selective impairment with reduced plasma IgE levels and BAL eosinophils (131). These Kit^W−sh/W−sh^ mice showing reduced AHR and inflammation compared to WT mice have the C57BL/6 genetic background. However, BALB/c-Kit^W−sh/W−sh^ mice showed as robust HDM-induced airway inflammatory phenotypes as did WT BALB/c mice (132). Allergic airway inflammation induced by repetitive HDM treatments was also reduced by cromoglygate, a mast cell stabilizer, before each HDM challenge (133). Interestingly, exacerbated AHR was induced by HDM in Kit^W−sh/W−sh^ mice engrafted with ST2-deficient mast cells, compared to Kit^W−sh/W−sh^ mice engrafted with WT mast cells (134), probably due to reduced prostaglandin E2 (PGE_2_) levels, which can inhibit IgE-mediated mast cell activation (135). IL-33/ST2-dependent mast cell induction of PGE_2_ could be responsible for the dampening effect on AHR (134). As researchers were concerned with abnormalities beyond mast cell deficiency in Kit mutant mast cell-deficient mice (e.g., Kit^W/Wv^ and Kit^W−sh/W−sh^ mice) and abnormal reconstitution of these mice with adoptively transferred mast cells (136, 137), non-Kit mutant mice have been used more often. Indeed, HDM-induced AHR was dependent on mast cells as demonstrated using Kit^W−sh/W−sh^ and Mas-TRECK mice (72).

Using wild-type and mutant BALB/c and FVB/N mice, McKnight et al. showed that neither IgE nor FcεRIα contributed to allergic airway disease, even in mice inoculated with the lowest dose of HDM, which readily induced detectable disease, but did not increase serum IgE or pulmonary mast cell levels (80). In contrast, high doses of HDM strikingly increased serum IgE and pulmonary mast cells, although both AHR and airway mast cell degranulation were equally elevated in wild-type and IgE-deficient mice. Thus, they concluded that IgE and FcεRIα-independent mechanisms are responsible for AHR and airway inflammation when low doses of HDM are inhaled repetitively. All the above HDM-induced airway inflammation data were obtained using soluble preparations of HDM. However, exacerbation of allergic asthma is also associated with an increase in ambient inhalable particulate matters (e.g., PM2.5 and PM10) from air pollutants. Furthermore, experimental allergic airway inflammation and/or AHR can be enhanced by particulate matters in an IL-33- or IL-1β-dependent manner (138–141). Importantly, Jin et al. compared AHR and pulmonary eosinophilia induced by soluble vs. particulate antigens (sAg vs. pAg) including HDM-conjugated polystyrene beads (142). They found that, compared with sAgs, pAgs triggered markedly heightened AHR and pulmonary eosinophilia in antigen-sensitized mice in a mast cell-dependent manner. pAgs mediated mast cell-dependent responses by enhancing retention of pAg/IgE/FcεRI complexes within lipid raft-enriched, CD63^+^ endocytic compartments, which prolonged IgE/FcεRI-triggered signaling and resulted in heightened cytokine responses. Animal models using particulate HDM are highly desirable to address the potential role of IgE/mast cells to mimic HDM-induced asthma.

### Animal Models of Rhinovirus-Induced Asthma Exacerbation

RV is a member of the enterovirus genus in the *Picornaviridae* family of small, non-enveloped positive strand RNA viruses (143). There are three human RV genotypes (A, B, C). Ninety percent of RV-A and RV-B use human intercellular adhesion molecule-1 (ICAM-1) as their receptor (144), while the minor group uses the human and mouse proteins of low-density lipoprotein receptor family (145). RV-C viruses use cadherin-related family member 3 (CDHR3) as their receptor (146). RV-C and RV-A cause severe respiratory illness more often than RV-B (147). RV-induced lower respiratory illness is increased in asthmatics and correlates with virus load, augmented Th2, and/or impaired Th1 and IL-10 immunity (148). RV-1B (a member of the minor receptor group of RV-A) infection of BALB/c mice or RV-16 (RV-A, a member of the major receptor group) infection of mouse-human ICAM-1 transgenic mice induces viral replication in airway epithelial cells, airway inflammation (neutrophilia and lymphocytosis), mucin secretion, and increased IFNs response, similarly to human RV infection (149). Compared to RV-A (RV-1B) infection, RV-C (RV-C15) infection induced higher BALF eosinophilia, mRNA expression of IL-5, IL-13, IL-25, Muc5ac and Gob5/Clca, protein production of IL-5, IL-13, IL-25, IL-33 and TSLP, and expansion of ILC2 in naive and HDM-immunized BALB/c mice (150). Eosinophilic inflammation and mRNA expression of IL-13, Muc5ac and Muc5b were ILC2-dependent. It is well-known that RV infection causes increased IgE levels (151) and RV-specific IgE (152). However, unlike RSV infection (153), no studies have tested if RV-specific IgE could sensitize mast cells for RV antigen-mediated activation and if RV causes IgE-dependent airway inflammation and AHR *in vivo*. Such experiments remain to be conducted.

Synergistic interactions between RV infection and allergen sensitization and exposure increase the risk of asthma exacerbations (154, 155). Toussaint et al. found that experimental infection of asthmatics with RV-16 induces double-stranded DNA (dsDNA) release in nasal lavages, which is correlated with type 2 immune-mediated asthma exacerbation severity (156). Then, by comparing immune responses between PBS-sensitized/HDM-challenged and HDM-sensitized/HDM-challenged mice infected with live or UV-inactivated RV-1B 1 day after the last (and the second) HDM challenge, they found that live RV-induced exacerbation is associated with a more robust type 2 immune response (increased eosinophils, lymphocytes in BALF); higher serum IgE; enhanced airway inflammatory cells and mucus production; AHR; increased Th2 cells in lung; stronger production of Th2 cytokines from lung and mediastinal lymph node cells; increased recruitment of monocyte-derived DCs to lung and mediastinal lymph nodes and cDC2 to lung and higher dsDNA release in the airways than the other groups (156). These responses were reduced by DNase treatment and mostly recapitulated by exogenous dsDNA in place of RV infection. DNase treatment reduced the recruitment of monocyte-derived DCs to lung and mediastinal lymph nodes. dsDNA releases induced by RV-16 in human patients and by RV-1B in the mouse model were part of neutrophil extracellular traps (NETs). The pathogenic role of NETosis in type 2 inflammation was revealed by neutrophil depletion and NET inhibitor.

## Discussion

Animal experiments have been the mainstay for mechanistic analysis of immune responses and disease pathogenesis, despite a wide recognition of differences between human and rodent immune systems (157–159). There is a discrepancy in clinical data showing omalizumab's efficacy in asthma and animal models lacking the effect of IgE or FcεRI deficiency. Clinical data indicate the presence of Th2-high and Th2-low asthmatic patients. Theoretically, Th2-high group may respond well to omalizumab. These different groups might be represented by animal models requiring IgE/FcεRI or not. However, things are not that simple. The discrepancy could be due to inappropriate animal models or simply lack of testing the role of IgE/FcεRI and mast cells. Most studies used a short course of less than a month. Careful analysis of acute vs. chronic models may reveal subtle differences in cellular and molecular requirements. Experiments with particulate HDM preparations or soluble HDM plus PM2.5 may be worth to test. Differences in HDM constituents between different lots could be a potential source of different results, as we experienced more than 10-fold differences in protein content per weight. Moreover, drastic differences in microbiota contained in HDM sources were recently noticed between different vendors (160). Thus, standardization of HDM preparations or use of single or combination of pure allergens is highly desirable. Incorporation of RV infection into HDM-induced airway inflammation adds another complexity. No systematic comparison between different RV types has been reported in airway disease or asthma exacerbation models, as experiments using RV-C has only recently begun. Since proliferation of human RV in mouse cells is severely limited, it may be useful to use a mouse cell-adapted RV variant (161). The landmark study by Toussaint et al. established an RV-induced asthma exacerbation model in HDM-allergic mice. Application or modification of this model to test effects of other RV species, mutant mice, and immunological/pharmacological agents will likely provide new insights into RV-induced asthma exacerbation in the future.

## Data Availability Statement

The original contributions presented in the study are included in the article/supplementary material, further inquiries can be directed to the corresponding author/s.

## Author Contributions

TK and YK wrote an initial manuscript. KK and TA edited it. All authors agree with the final version of the manuscript, contributed to the article, and approved the submitted version.

## Funding

Work in the TK lab is supported by the US National Institutes of Health grants R01 AI146042, R21 AI153867, UMI AI109565. TA is supported by the Ministry of Education, Culture, Sports, Science and Technology, Japan (20K08808) and by a Grant-in-Aid for Special Research in Subsidies for ordinary expenses of private schools from the Promotion and Mutual Aid Corporation for Private Schools of Japan.

## Conflict of Interest

The authors declare that the research was conducted in the absence of any commercial or financial relationships that could be construed as a potential conflict of interest.

## Publisher's Note

All claims expressed in this article are solely those of the authors and do not necessarily represent those of their affiliated organizations, or those of the publisher, the editors and the reviewers. Any product that may be evaluated in this article, or claim that may be made by its manufacturer, is not guaranteed or endorsed by the publisher.
